# Harmful Oral Bacilli and Their Relationship to Caries

**Published:** 1912-09-15

**Authors:** Theo. Von Beust


### HARMFUL ORAL BACILLI AND THEIR RELATIONSHIP TO CARIES.

BY DR. THEO. VON BEUST.

A Lecture given before the Berlin Dental Association.

Our knowledge of mouth organisms is, when one considers their role in dental biology, regrettably poor. Numerous attempts to obtain cultures have been made—it appears, however, so far, that the genuine mouth organisms cannot be artificially grown.

That the spirochaetes and the fusiform bacilli obtained by Muhlen, Baum Gartner, Paul and others are identical with the oral bacilli is by no means proven, and must for the present be left very much open to question.

Personally I very much doubt the identity, and I base my scepticism upon the Kreislauf pleomorph, the most important inhabitant of the mouth and one which the above-named cultivators have quite inexplicably neglected.

Plaut writes, after a study of the fusiform bacilli of the coating of the tongue: "When we observe the representatives of this group—which Miller has described under the name of 'Spirillum sputigenum'—dyed with ordinary aniline dyes, we can distinguish two different forms; bent rod-like structures, some with rounded and some with pointed extremities. The first have a sausage-like appearance, the second are shaped like crescent moons. When they are stirred seven different forms may be distinguished." Here, already, we may see that the existing classification is incomplete. The first reliable statements as to the forms found in the coating of the mouth are in Leber and Rottenstein. These experimenters have found on the mucous membrane, upon the tongue, between the teeth and in the coatings of Nasmyth's membrane at the onset of caries a fungus which they describe as the *Leptothrix buccalis.* The habit of this fungus is so well described that one can instantly distinguish it.

It seems almost as though Professor Miller were unwilling to take up the problem of the pleomorphic fructification of the oral bacilli, to which the work of Vicintini had again drawn attention. Miller drew up, in the eighties, a classification of the organisms to be found in the coating; its acceptance, however, of an already existing pleomorphology renders it comparatively valueless.

It is obvious that if experiments in the culture of oral bacilli are to be of use to us in our investigations of the oral deposits, this question must be cleared up.

With regard to the second part of my thesis, the relationship of oral bacilli to caries—we know as a result of recent researches that we cannot at present determine whether acids or ferments stand first in order of responsibility. That carbonic acid is related to caries was suggested as early as 1867. This relationship has since been reasserted in a variety of ways, and yet it seems to me that it has not so far been sufficiently substantiated. The net result of research into the physiology of plants leads us to conclude with Czepak that a whole series of organic acids—all those, that is to say, in which aluminium phosphate is soluble—have no share in the work of corrosion. If, then, we exclude them, there remain only carbonic, acetic, propionic, and butyric acid to be considered. The brown colorization of Congo red showed that in any case carbonic acid must come into the first rank in accounting for corrosion, since the other above-named acids produce a blue coloring. Jost thinks, however, that even if we admit carbonic acid as the agent we must not conclude that the roots cannot also secrete other substances, that plants are not able to draw more elements from the earth than can be set free by means of water containing carbonic acid.

The action of the algae in throwing off cells, a process analogous to that which one may observe in the roots of the higher plants and which is brought about by means of carbonic acid, may cast some light on our problem, and when one considers that all the bacteria so far discovered develop carbonic acid, and that carbonic acid is as much a product of putrefaction as of fermentation we may be justified in placing it side by side with lactic acid as a responsible factor in dental caries.—(Abstracted from a reprint of lecture in *Dental Record.*)

